# *Chlamydia trachomatis* Serology in Women with and without Ovarian Cancer

**DOI:** 10.1155/2008/219672

**Published:** 2008-12-18

**Authors:** Roberta B. Ness, Caixia Shen, Debra Bass, Carlynn Jackson, Kristen Moysich, Robert Edwards, Robert C. Brunham

**Affiliations:** ^1^School of Public Health, University of Texas, Houston, TX 77030, USA; ^2^UBC Centre for Disease Control, Vancouver, BC, Canada V5Z 4R4; ^3^Graduate School of Public Health and Pittsburgh Cancer Institute, University of Pittsburgh, Pittsburgh, PA 15261, USA; ^4^Department of Epidemiology, School of Public Health and Health Professions, University at Buffalo, Buffalo, NY 14214, USA; ^5^Magee-Women's Hospital, University of Pittsburgh Medical Center, Pittsburgh, PA 15213, USA

## Abstract

Pelvic inflammation has been implicated in the genesis of ovarian cancer. We conducted serologic measurements of *Chlamydia trachomatis* antibodies as a surrogate marker of chlamydial pelvic inflammatory disease. Women with ovarian cancer (*n* = 521) and population-based controls (*n* = 766) were tested. IgG antibodies to serovar D of chlamydia elementary bodies (EBs) were detected using an ELISA assay. The odds of having ovarian cancer among women with the highest titers (≥0.40 OD units) were 0.6 (95% CI 0.4–0.9). These data do not support our earlier finding of elevated titers for antibodies to *C. trachomatis* among women with ovarian cancer.

## 1. INTRODUCTION

Ovarian cancer is an often fatal disease with an uncertain etiology. We suggested that pelvic inflammation may play a role in the development of ovarian cancer [1]. PID has been linked to ovarian cancer risk in some [2, 3] but not all [4] studies. However, PID is poorly recalled in retrospective studies.

One quarter to three quarters of proven cases of PID are caused by *Neisseria gonorrhoeae* or *Chlamydia trachomatis* ascending into the upper genital tract to inflame the endometrium, tubes, and ovarian epithelium [5]. Of the two pathogens, *C. trachomatis* is the most common in American women [6].

 Chlamydia serology is a relatively specific marker of past chlamydial PID, particularly of more severe infections [7]. Its sensitivity is not complete; of women with chlamydial PID, about 60% will have antibodies to *C. trachomatis* [8] and among women with tubal factor infertility, a similar proportion will have IgG titers to chlamydia [9].

We previously reported pilot results from a population-based case-control study (117 cases and 170 controls) of ovarian cancer showing that ovarian cancer was significantly associated with high IgG antibody titers to chlamydia [10]. The purpose of the present study was to attempt to replicate this finding in a larger population-based case-control study of ovarian cancer.

## 2. MATERIALS AND METHODS

Subjects for this serologic analysis were part of a population-based case-control study conducted in a contiguous region comprising Western Pennsylvania, Eastern Ohio, and Southwestern New York State. Cases were residents of this geographic region with histologically confirmed, primary, epithelial ovarian, fallopian tube, or peritoneal cancer diagnosed between February 2003 and July 2006. Both invasive and borderline tumors were included. Women were referred from hospital tumor registries, clinical practices, or pathology databases and contacted with the permission of their gynecologists. Eligible women were at least 25 years of age and within 9 months of initial diagnosis.

Controls consisted of women at least age 25 who lived in telephone exchanges wherein cases resided. Random digit dialing was used to identify age-eligible women, and these were further screened by the study team to ensure that they had not had a previous oophorectomy or diagnosis of ovarian cancer. Eligible women were then invited to participate. Potential controls were frequency matched by 5-year age group and telephone exchange to cases in an approximately 2:1 ratio.

Women were interviewed in their homes by trained interviewers. The questionnaire included a reproductive and gynecological history, a contraceptive history, a medical history, a family history, and information on lifestyle practices.

We were able to draw blood on 92.5% of the interviewed cases and 84.4% of the interviewed controls. Blood samples were processed within 2 hours of collection by a laboratory technician. For this analysis, we selected the first 521 cases and 766 controls with complete questionnaires, tumor registry (e.g., histology) information, and adequate serum samples.

### 2.1. Serologic testing

Serologic testing for IgG antibodies to serovar D of *C. trachomatis* elementary bodies (EBs), the extracellular form of the chlamydia bacteria, was conducted in the reference laboratory of one of the authors (RB) using an ELISA technique. Final readings are based on a mean of duplicate runs. All assays were conducted by personnel masked to case/control status. The intra-assay coefficient of variation for chlamydia antibodies was 0.06, representing excellent intra-assay replication. Among masked replicates admixed into the test set, Pearson correlation coefficients were 0.90 for chlamydia, again representing excellent interassay variability.

### 2.2. Statistical analysis

Each of the antibody levels tested was measured in optical density (OD) units (range 0.0–0.4+). We log transformed all OD units to reduce skewing when considering these as continuous measures and categorized OD units into neat whole number categories when considering these as discrete measures. These cut points corresponded to those in our published pilot study [10]. Odds ratios, with corresponding 95 percent confidence intervals, were calculated as the primary measure of effect size. Odd ratios were adjusted in unconditional logistic regression models for any residual effect of age and for family history of ovarian cancer in any first degree relative (yes/no), tubal ligation (yes/no), nulliparity versus any parity, years of oral contraception (continuous), and education (<high school/high school or equivalent/>high school). In secondary analyses, we divided serologic titers by quartiles; we also performed secondary analysis limiting our evaluation to only women with invasive ovarian cancer. We also stratified by age to assess cohort and age effects. Chlamydial infections typically occur in younger women. Older women may have time-related diminished antibody titers.

## 3. RESULTS

About one quarter of women were younger than the age of 50 and half were age 50 to 65 (see Table 1). Only about 1% of women reported having PID and 4% reported having gonorrhea or chlamydia. The well-established protection against ovarian cancer afforded by oral contraception, pregnancies, and tubal ligation, and the risk from a family history of ovarian cancer were demonstrated here.

After adjustment for possible confounding factors and based on our previous antibody titer cut points [10], women with ovarian cancer were less likely than controls to have high chlamydia EB OD unit titers (≥0.40 versus <0.10) (OR 0.6, 95% CI 0.4–0.9; test for trend *P* = .001) (see Table 2). We then recategorized chlamydia EB OD units as quartiles based on the distribution of antibody values among controls (see Table 3). After adjustment for covariates, the highest quartile of chlamydia EB antibodies was inversely nonsignificantly associated with ovarian cancer (OR 0.7, 95% confidence interval 0.5–1.1).

In age-stratified analyses, we continued to find the chlamydia antibodies that reduced ovarian cancer risk (see Table 4). Results were also similar in analyses limited to invasive ovarian cancer; the risk for ovarian cancer among women in the highest quartile of chlamydia EB antibodies versus the lowest was 0.6 (0.4–0.9, test for trend *P* = .021).

## 4. DISCUSSION

We found that women with ovarian cancer were less likely to have high levels of IgG to *C. trachomatis* serovar D EBs. Our data are inconsistent with our previously published pilot results [10] which showed higher serologic titers for *C. trachomatis* among ovarian cancer patients.

Our current finding is the reverse of the link between chlamydia and ovarian cancer in our previous analysis [10]. This is difficult to explain. The same laboratory conducted serologic analyses using similar methods for both studies. A population-based case-control method was used to recruit women into both ovarian cancer case-control studies.

Explanations for a lack of association include the long period between exposure to chlamydia (in the reproductive period) and blood collection (generally after the age of 50) in our population. If titers fall significantly over time, they may have become undetectable in some women. Data from a Dutch study suggested that 18% of women had a decline of twofold in chlamydia titers over four years [11]. However in our own data, over 5–7 years of followup, women with the highest antibody titers rarely moved to the lowest tertile over time [12].

Another explanation is the lack of sensitivity of antichlamydial antibody serologic testing. Only about 60% of women with PID develop detectable serology, and the test does not detect gonorrhea, another cause of PID. This lack of sensitivity would have resulted in erroneously missing some women with prior PID and would have resulted in an odds ratio biased toward the null. Moreover, it is possible that ovarian cancer itself or its treatment might acutely reduce chlamydia titers, and therefore mask an association.

Strengths of this study include the population-based ascertainment of cases and controls, the standardized collection and storage of bloods, the measurement of chlamydia EB antibodies at a reference, research laboratory, with laboratory personnel masked to case-control status, and evidence of an independent effect after adjustment for potentially confounding factors.

In summary, our findings do not support previous evidence of a link between chronic persistent chlamydia infection, the most common cause of PID, and risk for ovarian cancer.

## Figures and Tables

**Table 1:** Frequencies of demographic and reproductive characteristics by case/control status.

Variable	Case subjects no. (%)	Control subjects no. (%)	*χ* ^2^ (*P* value)
Age group, years			
24–49	135 (25.9)	204 (26.6)	9.29 (.026)
50–56	111 (21.3)	208 (27.2)	
57–66	129 (24.8)	187 (24.4)	
≥67	146 (28.0)	167 (21.8)	

Ethnic group			
White	495 (95.0)	736 (96.1)	1.14 (.57)
Black	19 (3.6)	20 (2.6)	
Other	7 (1.3)	10 (1.3)	

Education			
Less than high school	46 (8.8)	37 (4.8)	13.56 (.001)
High school	180 (34.5)	229 (29.9)	
Posthigh school	295 (56.6)	500 (65.3)	

Family history of ovarian cancer			
No	487 (95.3)	731 (97.3)	3.74 (.053)
Yes	24 (4.7)	20 (2.7)	

Live births			
None	124 (23.8)	104 (13.6)	22.23 (.000)
Any	397 (76.2)	662 (86.4)	

Tubal ligation			
No	389 (78.3)	490 (65.0)	25.3 (.000)
Yes	108 (21.7)	264 (35.0)	

Oral Contraception, years			
0	223 (42.8)	214 (27.9)	40.02 (.000)
<1–4	205 (39.3)	321 (41.9)	
5–9	59 (11.3)	134 (17.5)	
≥10	34 (6.5)	97 (12.7)	

Menopausal status			
Premenopausal	142 (30.4)	194 (29.9)	.028 (.87)
Postmenopausal	325 (69.6)	454 (70.1)	

Self-report PID			
No	515 (98.8)	759 (99.1)	.18 (.68)
Yes	6 (1.2)	7 (0.9)	

Self-report gonococcal or chlamydial cervicitis			
No	502 (96.4)	736 (96.1)	.062 (.80)
Yes	19 (3.6)	30 (3.9)	

**Table 2:** Frequencies and adjusted odds ratios (ORs) and 95% confidence intervals (CIs) for chlamydial elementary bodies optical density (OD) units, in cases and controls, categorized by previously defined cut points. (ORs were adjusted for age, education, family history of ovarian cancer, tubal ligation, nulliparity/any parity, and years of oral-contraceptive use).

	Chlamydia EB		

OD units	Case subjects	Control subjects	OR (95% CI)
<0.10	248	342	1.0
0.10–0.199	138	173	1.1 (0.8–1.5)
0.20–0.399	73	113	0.9 (0.6–1.2)
≥0.40	62	138	0.6 (0.4–0.9)

**Table 3:** Frequencies and adjusted odds ratios (ORs) and 95% confidence intervals (CIs) for chlamydial elementary bodies, categorized by quartiles, in case and control subjects. (ORs were adjusted for age, education, family history of ovarian cancer, tubal ligation, nulliparity/any parity, and years of oral-contraceptive use.)

	Chlamydia EB		

EB quartiles	Case subjects	Control subjects	OR (95% CI)
<0.06	133	189	1.0
0.06–0.11	133	189	1.1 (0.8–1.5)
0.11–0.24	146	176	1.2 (0.9–1.7)
≥0.25	109	212	0.7 (0.5–1.1)

**Table 4:** Frequencies and adjusted odds ratios (ORs) and 95% confidence intervals (CIs) for chlamydial elementary bodies optical-density (OD) units, in case and control subjects stratified by age.

	OD units	Case subjects	Control subjects	OR (95% CI)
Age 24–49	<0.10	69	82	1.0
	0.10–0.199	32	42	0.9 (0.5–1.6)
	0.20–0.399	18	37	0.6 (0.3–1.1)
	≥0.40	16	43	0.4 (0.2–0.9)

				Trend = .007

Age 50–56	<0.10	55	103	1.0
	0.10–0.199	22	47	0.9 (0.5–1.6)
	0.20–0.399	15	20	1.4 (0.7–3.0)
	≥0.40	19	38	0.9 (0.5–1.8)

				Trend = .908

Age 57–66	<0.10	58	88	1.0
	0.10–0.199	41	38	1.6 (0.9–2.8)
	0.20–0.399	19	31	0.9 (0.5–1.8)
	≥0.40	11	30	0.6 (0.3–1.2)

				Trend = .227

Age ≥67	<0.10	66	69	1.0
	0.10–0.199	43	46	1.0 (0.6–1.7)
	0.20–0.399	21	25	0.9 (0.4–1.7)
	≥0.40	16	27	0.6 (0.3–1.3)

				Trend = .217
